# Safety and Efficacy of Roxadustat for Anemia in Patients With Chronic Kidney Disease: A Meta-Analysis and Trial Sequential Analysis

**DOI:** 10.3389/fmed.2021.724456

**Published:** 2021-08-31

**Authors:** Chao Liu, Zhangning Fu, Jiawei Jiang, Kun Chi, Xiaodong Geng, Zhi Mao, Chengcheng Song, Guannan Sun, Quan Hong, Guangyan Cai, Xiangmei Chen, Xuefeng Sun

**Affiliations:** ^1^Department of Nephrology, Chinese PLA General Hospital, Chinese PLA Institute of Nephrology, State Key Laboratory of Kidney Diseases, National Clinical Research Center for Kidney Diseases, Beijing, China; ^2^Department of Critical Care Medicine, Tianjin Medical University First Center Clinical College, Tianjin, China; ^3^Department of Critical Care Medicine, Chinese PLA General Hospital, Beijing, China

**Keywords:** roxadustat, anemia, chronic kidney disease, meta-analysis, trial sequential analysis

## Abstract

**Background:** Roxadustat, a hypoxia-inducible factor prolyl-hydroxylase inhibitor (HIF-PHI), has been used to treat anemia in patients with chronic kidney disease (CKD). However, its safety and efficacy remain controversial.

**Methods:** The PubMed, EMBASE, Science Citation Index, Cochrane Central Register of Controlled Trials, and Clinical Trial Registries databases were searched for relevant studies published up to April 2021. We identified randomized controlled trials (RCTs) comparing roxadustat with placebo or erythropoiesis-stimulating agents (ESAs) in anemia patients with CKD with or without dialysis.

**Results:** Eleven studies including 6,631 patients met the inclusion criteria. In non-dialysis-dependent (NDD-) and dialysis-dependent (DD-) CKD patients, the total adverse events were not significantly different between the roxadustat and control (placebo for NDD-CKD patients and ESA for DD-CKD patients) groups [relative risk (RR) = 1.02, 95% confidence interval (CI) = 1.00, 1.04, *P* = 0.08, and RR = 1.22, 95% CI = 0.91, 1.64, *P* = 0.18, respectively], and the trial sequential analysis (TSA) confirmed the result in the NDD-CKD groups. No significant differences in hyperkalemia and infection incidences were found between roxadustat and placebo in the DD-CKD groups. The pooled results showed that roxadustat significantly increased the hemoglobin response rate compared with placebo in the NDD-CKD group and had an effect similar to that of ESA in the DD-CKD group. However, iron metabolism parameters did not seem to be obviously optimized by roxadustat.

**Conclusion:** Roxadustat can be safely used in CKD patients. Oral roxadustat was more effective than placebo as a therapy for anemia in NDD-CKD patients and non-inferior to ESA in correcting anemia in DD-CKD patients. However, additional clinical trials are still needed to further prove whether roxadustat can optimize iron metabolism.

## Introduction

Anemia is a common complication in millions of patients with chronic kidney disease (CKD) and is associated with increased morbidity and mortality in dialysis-dependent (DD-) and non-dialysis-dependent (NDD-) CKD patients (1).

Erythropoiesis-stimulating agents (ESAs) and adjuvant intravenous (IV) iron supplementation represent the current standard of care for DD-CKD patients with anemia (2). However, some safety concerns still exist. For example, there is evidence that hemoglobin normalization by ESAs may have no benefit in CKD patients and may be associated with increased rates of stroke, hypertension, cardiovascular events and mortality (3–5). Iron supplementation also has some significant drawbacks. IV iron also has the potential to stimulate bacterial growth, increasing the risk of infection, and causing direct cellular toxicity (6, 7). Total body iron overload increases hepcidin levels, which is likely to contribute to the incidence and severity of anemia and may cause ESA resistance, resulting in the need for increased doses of ESAs to achieve target hemoglobin levels and may consequently increase the rate of adverse events (8, 9).

Recently, a new therapeutic class of agents, hypoxia-inducible factor prolyl-hydroxylase inhibitors (HIF-PHIs), which can restore endogenous erythropoietin (EPO) production and may optimize iron metabolism, has been approved in China and Japan for the treatment of anemia in CKD patients (10–12). Roxadustat, as a type of HIF-PHI, was evaluated for safety and efficacy in seven recent phase 3 clinical trials (13–19). Therefore, we performed this meta-analysis and trial sequential analysis (TSA) to further evaluate the safety and efficacy of roxadustat for the treatment of anemia in CKD patients.

## Methods

The Preferred Reporting Items for Systematic Reviews and Meta-Analyses (PRISMA statement) guidelines were used to perform this meta-analysis (20). This meta-analysis was prospectively registered on PROSPERO database (Registration number: CRD42020160014).

### Data Sources and Study Selection

The PubMed, EMBASE, Science Citation Index, Cochrane Central Register of Controlled Trials, and Clinical Trial Registries databases were searched for relevant studies published up to April 2021. The search terms included “FG-4592,” “roxadustat,” “anemia,” “chronic kidney disease,” “chronic kidney failure,” “chronic renal insufficiency,” “chronic renal disease,” and “randomized controlled trial.” The search was limited to studies involving human subjects, and no language restrictions were applied. The citations of the included studies were scanned to identify additional relevant studies.

### Inclusion and Exclusion Criteria

The inclusion criteria were as follows: (1) study design: randomized controlled trial (RCT); (2) population: anemia patients with CKD (>18 years old) with or without dialysis; (3) intervention: roxadustat compared with placebo or ESA (epoetin alfa or darbepoetin alfa); and (4) primary outcome: the safety of roxadustat. The secondary outcomes included the hemoglobin response rate, the change in hemoglobin, hepcidin, transferrin saturation, ferritin, total iron-binding capacity and total iron during treatment. The exclusion criteria were as follows: (1) studies that involved healthy individuals; (2) studies that included inappropriate comparisons or did not include a reference group; and (3) studies with research data that could not be extracted and analyzed.

### Data Extraction and Quality Assessment

Two reviewers (CL and ZF) independently extracted the data using a standardized, pre-established form. If one study contained more than one clinical trial (trials with national clinical trial numbers), we extracted the data from the study separately. Each trial was assessed using the Cochrane risk of bias tool. The standard criteria included the following domains: random sequence generation, allocation concealment, blinding of participants and personnel, blinding of outcome assessment, incomplete outcome data, selective reporting and other bias. Any disagreements were resolved by a third reviewer.

### Data Synthesis and Statistical Analysis

All data were analyzed using Review Manager (version 5.3) and R software (version 3.5.1). The effect size was assessed by relative risks (RRs) with 95% confidence intervals (CIs) for dichotomous outcomes and mean differences (MDs) with 95% CIs for continuous outcomes. Subgroup analyses were conducted to explore between-study heterogeneity. Publication bias was assessed using Begg's and Egger's tests. All *P*-values were two-sided, and a *P* < 0.05 indicated a statistically significant difference.

### Trial Sequential Analysis

In the meta-analyses, trial sequential analysis (TSA) was used to reduce the risk of reaching a false-positive or false-negative conclusion (21). When the cumulative Z-curve crossed the trial sequential monitoring boundary or entered the futility area, a sufficient level of evidence for the anticipated intervention effect was reached, and no further trials were needed. If the Z-curve did not cross any of the boundaries and the required information size (RIS) had not been reached, evidence to reach a conclusion was insufficient, and more trials were needed to confirm the results (22). For this TSA, we estimated the RIS based on an RR reduction (RRR) of 20%. The type I error (α) = 0.05 (two-sided) and power (1 – β) = 0.80. The control event proportion was calculated from the comparator group (23). The TSA was conducted using TSA Version 0.9.5.10 Beta (www.ctu.dk/tsa).

## Results

### Study Selection and Study Characteristics

**Figure 1:**
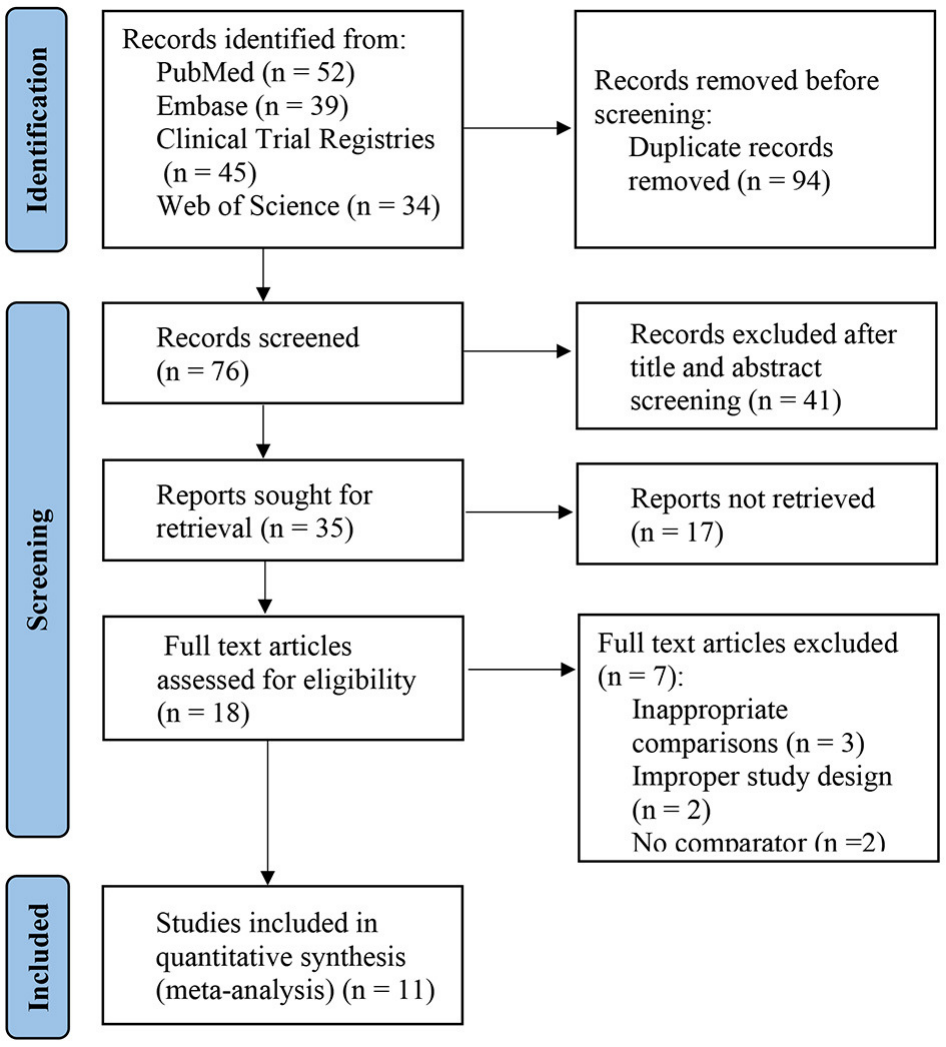
Flow chart of the study selection.

The study retrieval process is outlined in Figure 1. In total, eleven studies (13–19, 24–27) (containing 12 clinical trials) including 6,631 patients met the inclusion criteria. The study characteristics are summarized in Table 1. These studies were published between 2015 and 2021. Seven studies (13–19) were phase 3 clinical trials, and the others were phase 2 clinical trials. Three studies (13, 14, 25) were conducted in the Chinese population, two (26, 27) in the American population and two (15, 24) in the Japanese population, while the others were multicenter studies (16–19). In NDD-CKD patients, the interventions were roxadustat vs. a placebo. In DD-CKD patients, the interventions were roxadustat vs. ESA (epoetin alfa or darbepoetin alfa).

**Table 1:** Characteristics of the included studies.

**Source**	**Country**	**NCT number**	**Setting**	**Intervention**	**Type of patients**	**Duration, weeks**	**Iron therapy**	**No. of patients (M/F)**	**Mean age, years**
Akizawa et al. (24)	Japan	01964196	Phase 2, randomized, parallel-group, double-blind, placebo-controlled	T: Roxadustat (50, 70, or 100 mg) TIW for 6 weeks followed by dose adjustments to maintain Hb at 10–12 g/dl for 18 weeksC: Placebo	NDD-CKD	24	Oral	T: 80(39/41)C: 27(11/16)	T: 64.4 ± 8.7^a^ C: 61.9 ± 10.6^a^
Chen et al. (25)	China	01599507	Phase 2, randomized, double-blind, placebo-controlled	T: Roxadustat (1.1-1.75 mg/kg, 1.5-2.25 mg/kg) TIW for 8 weeksC: Placebo	NDD-CKD	8	Oral	T: 61(18/43)C: 30(8/22)	49.7 ± 13.2^a^
Besarab et al. (27)	United States	00761657	Phase 2, randomized, single-blind, placebo-controlled	T: Roxadustat (0.7,1.0, 1.5, 2.0 mg/kg) BIW or TIW for 6 weeksC: Placebo	NDD-CKD	6	Oral	T: 88(33/55)C: 28(16/12)	T: 64.0 (47-82)^b^ C: 68.6 (56-79)^b^
Chen et al. (13)	China	02652819	Phase 3, double-blind, placebo-controlled	T: Roxadustat (70 or 100 mg) TIW for 8 weeksC: Placebo	NDD-CKD	8	Oral	T: 101(36/65)C: 51(20/31)	T: 54.7 ± 13.3^a^ C: 53.2 ± 13.1^a^
Coyne et al. (16)	163 sites	01750190	Phase 3, randomized, double-blind, placebo-controlled	T: Roxadustat (70 or 100 mg) TIW for 52 weeksC: Placebo	NDD-CKD	52	Oral	T: 616(241/375)C: 306(130/176)	T: 64.9 ± 12.6^a^ C: 64.8 ± 13.2^a^
Fishbane et al. (17)	385 centers (25 countries)	02174627	Phase 3, multicenter, randomized, double-blind, placebo-controlled	T: Roxadustat (70 mg) TIW for 52 weeksC: Placebo	NDD-CKD	52	Oral	T: 1384(564/820)C: 1377(603/774)	T: 60.9 ± 14.7^a^ C: 62.4 ± 14.1^a^
Shutov et al. (19)	138 sites	01887600	phase 3, multicenter, randomized, double-blind, placebo-controlled	T: Roxadustat (70 or 100 mg) TIW for at least 52 weeks up to a maximum of 104 weeksC: Placebo	NDD-CKD	52-104	Oral	T: 391(169/222)C: 203(99/104)	T: 62.0 (20-89)^b^ C: 63.0 (26-90)^b^
Akizawa et al. (15)	Japan	02952092	Phase 3, multicenter, randomized, double-blind, active-comparator	T: Roxadustat (70 or 100 mg) TIW for 24 weeksC: Darbepoetin alfa	DD-CKD	24	Oral	T: 150(101/49)C: 151(107/44)	T: 64.6 ± 11.7^a^ C: 64.9 ± 10.1^a^
Chen et al. (25)	China	01596855	Phase 2, randomized, open-label active-comparator	T: Roxadustat (1.1-1.8 mg/kg, 1.5-2.3 mg/kg, 1.7-2.3 mg/kg) TIW for 8 weeksC: Epoetin alfa	DD-CKD	6	Oral	T: 74(45/29)C: 22(13/9)	50.8 ± 12.6^a^
Provenzano et al. (26)	United States	01147666	Phase 2, randomized, multicenter, open-label, consecutive-cohort, multidose study	T: Roxadustat (1.0, 1.5, 1.8, or 2.0 mg/kg) TIW for 6 weeksC: Epoetin alfa	DD-CKD	6	Oral	T: 41(27/14)C: 13(9/4)	T: 55.8 ± 13.4^a^ C: 59.5 ± 10.1^a^
				T: Roxadustat (adjust dose according to part 1) TIW for 19 weeksC: Epoetin alfa	DD-CKD	19	Oral	T: 67(45/22)C: 23(14/9)	T: 56.9 ± 12.1^a^ C: 57.0 ± 11.6^a^
Chen et al. (14)	China	02652806	Phase 3, randomized, open-label, active-controlled	T: Roxadustat (100 or 120 mg) TIW for 26 weeksC: Epoetin alfa	DD-CKD	26	Oral	T: 204(126/78)C:100 (58/42)	T: 47.6 ± 11.7^a^ C: 51.0 ± 11.8^a^
Provenzano et al. (18)	19 countries	02052310	phase 3, multicenter, randomized, open-label, active-controlled	T: Roxadustat (70 or 100 mg) TIW for 52 weeksC: Epoetin alfa	DD-CKD	52	Oral	T: 522(309/213)C: 521(307/214)	T: 53.8 ± 14.7^a^ C: 54.3 ± 14.6^a^

*BIW, twice weekly; C, control group; CKD, chronic kidney disease; DD, dialysis-dependent; Hb, hemoglobin; M/F, male/female; NCT, National Clinical Trial; NDD, non-dialysis-dependent; T, treatment group; TIW, three times weekly;*

a *Mean±standard deviation;*

b *Median (interquartile range)*.

### Quality Assessment of the Included Studies

**Figure 2:**
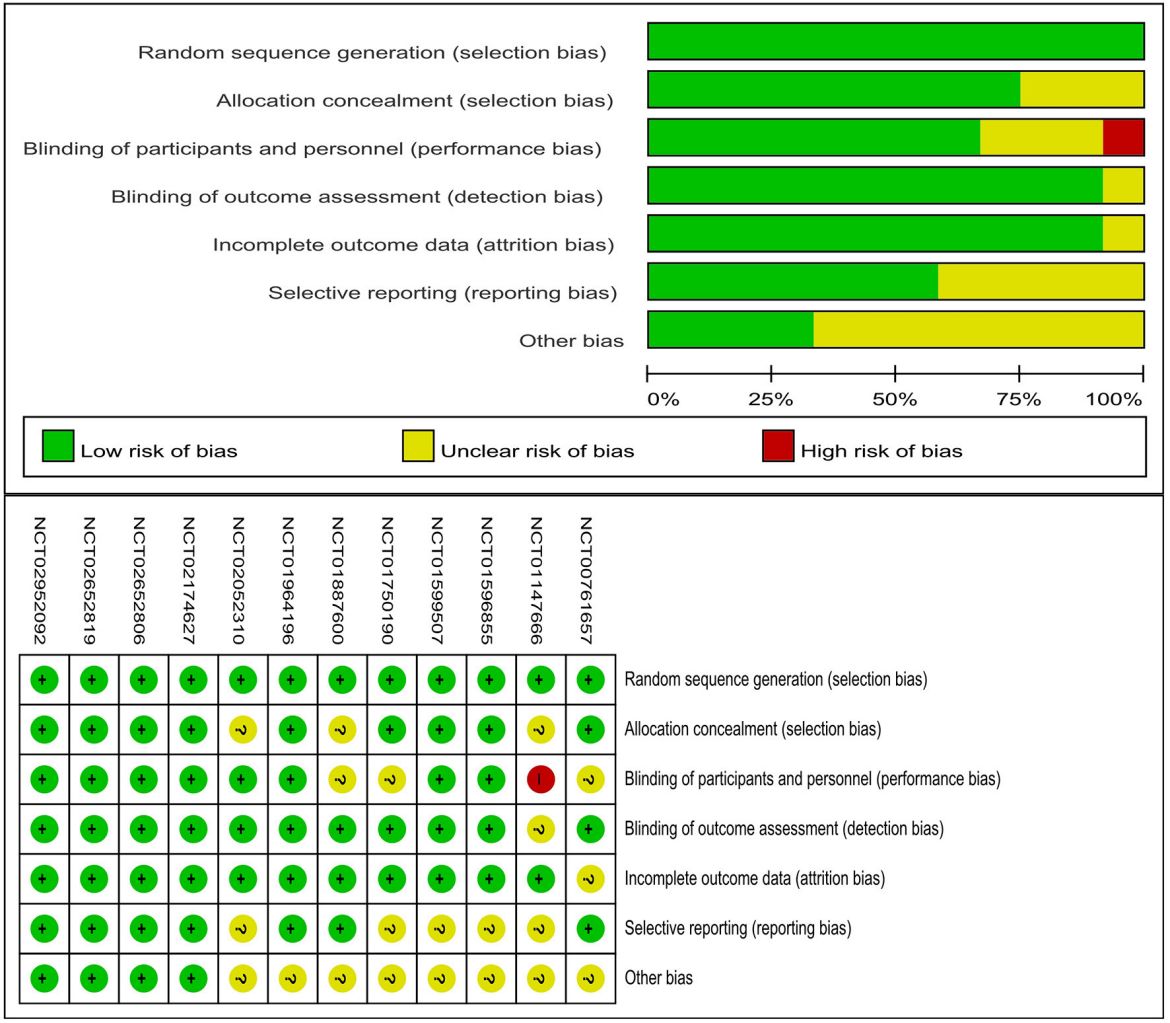
Assessment of risk of bias. NCT, National Clinical Trial.

The details of the risk of bias tool are shown in Figure 2. All studies had a low risk of selection bias. Randomized sequence generation and allocation concealment were reported adequately in most studies. Four studies were high-quality studies with a low risk of bias for all items.

### Safety of Roxadustat

**Figure 3:**
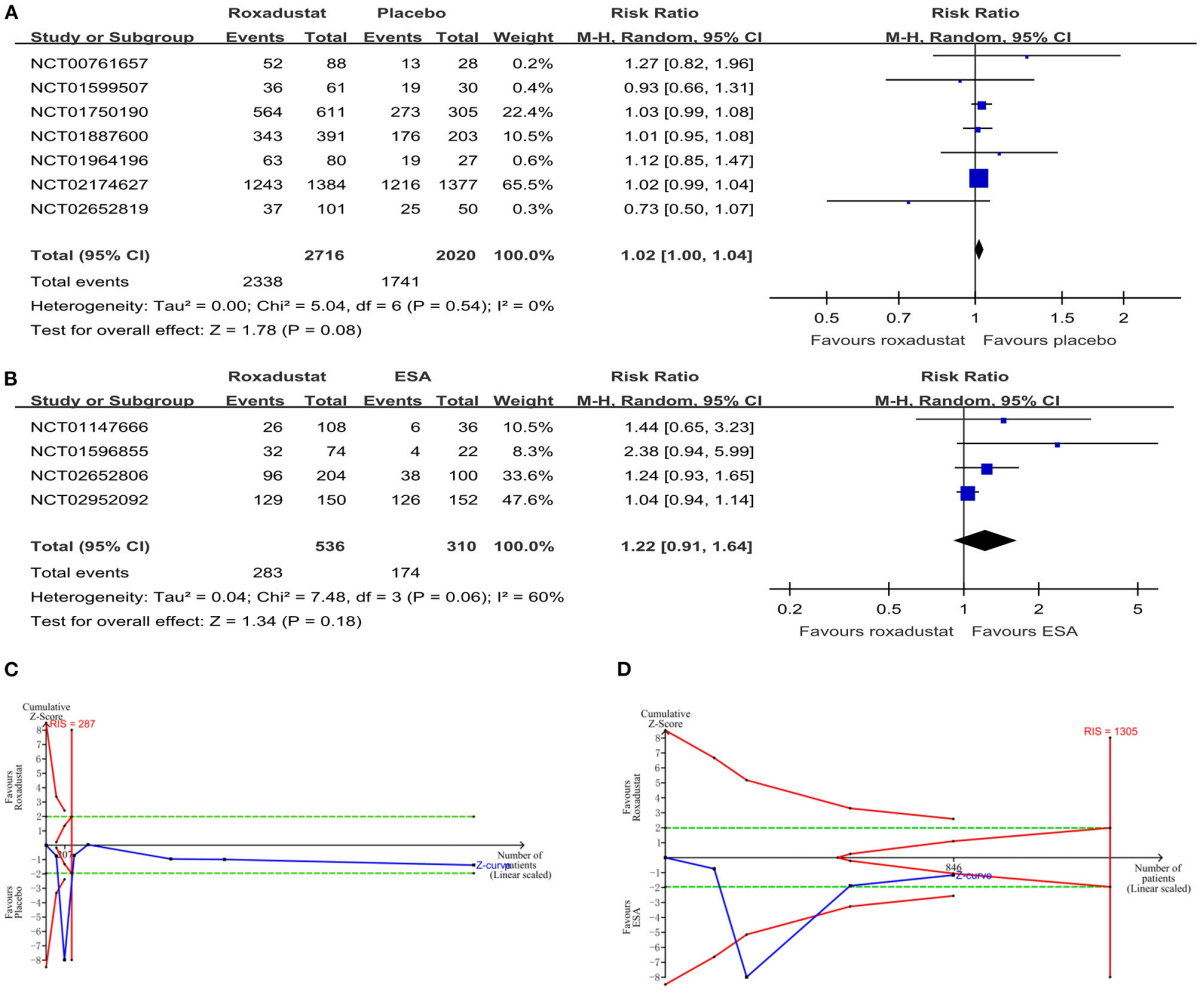
The safety of roxadustat. **(A)** Forest plot of the safety of roxadustat and the placebo in NDD-CKD patients. **(B)** Forest plot of the safety of roxadustat and epoetin alfa in DD-CKD patients. **(C)** Random effects model of the TSA of safety of roxadustat and the placebo in NDD-CKD patients. A diversity-adjusted information size of 287 participants was calculated based on an adverse event rate of 86.2% in the placebo group and a relative risk reduction (RRR) of 20%, with α = 5% (two-sided), β = 20%, and I^2^ = 0%. The solid blue line represents the cumulative Z-curve, which crossed the futility boundary (solid red line). **(D)** Random effects model of the TSA of safety of roxadustat and ESA in DD-CKD patients. A diversity-adjusted information size of 1,305 participants was calculated based on an adverse event rate of 56.1% in the ESA group and an RRR of 20%, with α = 5% (two-sided), β = 20%, and I^2^ = 60%. The solid blue line represents the cumulative Z-curve, which crossed the conventional boundary (dashed green line) but did not cross the trial sequential monitoring boundary (solid red line). CKD, chronic kidney disease; CI, confidence interval; DD, dialysis-dependent; ESA, erythropoiesis-stimulating agents; NDD, non-dialysis-dependent.

All studies reported adverse events during the treatment process. In the NDD-CKD patient groups (13, 16, 17, 19, 24, 25, 27), the results showed that there was no significant difference in the total adverse events between the roxadustat and placebo groups (RR = 1.02, 95% CI = 1.00, 1.04, *P* = 0.08, *I*^2^ = 0%; Figure 3A), and the TSA confirmed this result [the cumulative Z-curve crossed the futility boundary and entered the futility area; relative risk reduction (RRR) = 20%, α = 5%, β = 20%, *I*^2^ =0%; Figure 3C]. In the DD-CKD patient groups (14, 15, 25, 26), the results showed that the frequency of total adverse events was also similar between the roxadustat and ESA treatment groups (RR = 1.22, 95% CI = 0.91, 1.64, *P* = 0.18, *I*^2^ = 60%; Figure 3B), but the TSA did not confirm this result (the cumulative Z-curve just crossed the conventional boundary but did not cross the trial sequential monitoring boundary; RRR = 20%, α = 5%, β = 20%, *I*^2^ = 60%; Figure 3D). No significant differences in hyperkalemia and infection incidences were found in the DD-CKD patient groups (Table 2). However, there was an increased risk of hyperkalemia in the roxadustat group vs. the placebo group in NDD-CKD patients (RR = 1.24, 95% CI = 1.02, 1.51, *P* = 0.03, *I*^2^ = 0%, Table 2).

**Table 2:** Other outcomes.

**Outcomes**	**Subgroup**	**Comparison**	**Number of studies**	**Risk ratio or Mean difference (95%CI)**	**Test for effect (*p* value)**	**HeterogeneityI^**2**^ (*p* value)**
Hyperkalemia	NDD-CKD	Roxadustat vs. Placebo	7	1.24 [1.02, 1.51]	0.03	0% (0.89)
	DD-CKD	Roxadustat vs. ESA	4	0.63 [0.04, 9.74]	0.74	78% (0.01)
Any infection	NDD-CKD	Roxadustat vs. Placebo	7	1.05 [0.85, 1.31]	0.64	47% (0.08)
	DD-CKD	Roxadustat vs. ESA	5	1.21 [0.95, 1.54]	0.12	0% (0.81)
Cardiovascular disorders	NDD-CKD	Roxadustat vs. Placebo	6	1.08 [0.84, 1.40]	0.55	30% (0.21)
	DD-CKD	Roxadustat vs. ESA	5	1.08 [0.84, 1.38]	0.56	0% (0.52)
Oral iron therapy rate	NDD-CKD	Roxadustat vs. Placebo	2	1.23 [0.79, 1.92]	0.35	0% (0.43)
	DD-CKD	Roxadustat vs. ESA	3	0.93 [0.78, 1.10]	0.39	34% (0.22)
ΔHemoglobin (g/dL)	NDD-CKD	Roxadustat vs. Placebo	6	1.65 [1.37, 1.93]	<0.01	92% (<0.01)
	DD-CKD	Roxadustat vs. ESA	4	0.16 [0.05, 0.27]	<0.01	0% (0.44)
ΔHepcidin (ng/mL)	NDD-CKD	Roxadustat vs. Placebo	4	−19.16 [−37.67, −0.65]	0.04	96% (<0.01)
	DD-CKD	Roxadustat vs. ESA	4	−13.62 [−35.77, 8.53]	0.23	77% (<0.01)
ΔFerritin (ng/mL)	NDD-CKD	Roxadustat vs. Placebo	6	−50.16 [−71.19, −29.13]	<0.01	55% (0.05)
	DD-CKD	Roxadustat vs. ESA	5	−17.46 [−61.63, 26.71]	0.44	73% (<0.01)
ΔTotal iron-binding capacity (μg/dL)	NDD-CKD	Roxadustat vs. Placebo	4	70.22 [33.86, 106.58]	<0.01	94% (<0.01)
	DD-CKD	Roxadustat vs. ESA	4	42.56 [22.95, 62.18]	<0.01	86% (<0.01)
ΔTransferrin saturation (%)	NDD-CKD	Roxadustat vs. Placebo	6	−2.73 [−5.23, −0.23]	0.03	74% (<0.01)
	DD-CKD	Roxadustat vs. ESA	5	0.62 [−0.69, 1.94]	0.35	0% (0.68)
ΔTransferrin (g/L)	NDD-CKD	Roxadustat vs. Placebo	3	0.65 [0.26, 1.04]	<0.01	95% (<0.01)
	DD-CKD	Roxadustat vs. ESA	3	0.42 [0.29, 0.54]	<0.01	75% (0.02)
ΔTotal iron (μg/dL)	NDD-CKD	Roxadustat vs. Placebo	3	−2.21 [−9.24, 4.81]	0.54	20% (0.29)
	DD-CKD	Roxadustat vs. ESA	4	16.28 [9.39, 23.17]	<0.01	27% (0.25)
ΔLDL cholesterol (mg/dL)	NDD-CKD	Roxadustat vs. Placebo	3	−16.11 [−32.15, −0.07]	0.05	98% (<0.01)
	DD-CKD	Roxadustat vs. ESA	2	−18.59 [−21.77, −15.42]	<0.01	0% (0.77)

*CKD, chronic kidney disease; CI, confidence interval; DD, dialysis-dependent; ESA, erythropoiesis-stimulating agents; NA, not applicable; NDD, non-dialysis-dependent*.

### Hemoglobin Response Rate

**Figure 4:**
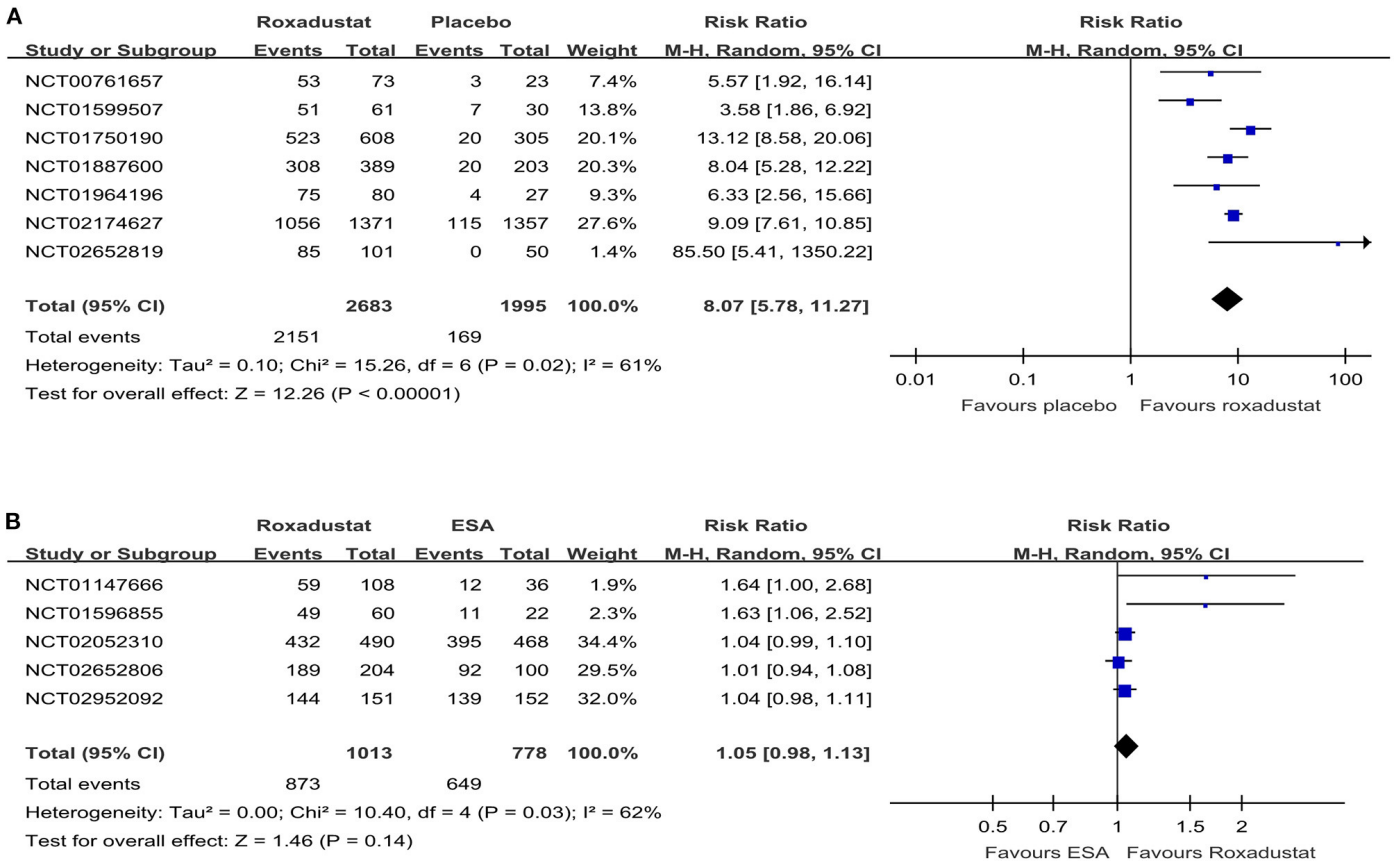
Forest plot of the hemoglobin response rate. **(A)** Forest plot of the hemoglobin response rates of roxadustat and the placebo in NDD-CKD patients. **(B)** Forest plot of the hemoglobin response rates of roxadustat and ESA in DD-CKD patients. CKD, chronic kidney disease; CI, confidence interval; DD, dialysis-dependent; ESA, erythropoiesis-stimulating agents; NDD, non-dialysis-dependent.

The hemoglobin response rate was defined as the percentage of patients who had a response to a trial regimen, which was defined as an increase from baseline of at least 1.0 g per deciliter in the hemoglobin level (13). All the included studies reported the hemoglobin response rate. The pooled results showed that roxadustat significantly increased the hemoglobin response rate compared with the placebo in the NDD-CKD patient group (RR = 8.07, 95% CI = 5.78, 11.27, *P* < 0.01, *I*^2^ = 61%; Figure 4A), and it had an effect similar to that of ESA in the DD-CKD patient group (RR =1.05, 95% CI = 0.98, 1.13, *P* = 0.14, *I*^2^ = 62%; Figure 4B).

### Effects on Hemoglobin

We used the hemoglobin change from baseline to evaluate the effect of roxadustat on hemoglobin. Nine studies (13–19, 24, 25) reported comparisons of hemoglobin. If the studies reported the data for more than one dose, we chose the optimal dose (the most effective dose with the lowest risk of adverse events), such as the high dose (1.50–2.25 mg/kg) in the Chen et al. study (2017) (25), 100 mg TIW in the Akizawa et al. study (24) and mid-range dose (1.5–2.3 mg/kg) in the study of Chen et al. (25) in the roxadustat group to perform the pooled analysis. Among both the NDD-CKD and DD-CKD patients, the hemoglobin change from baseline was significantly increased in the roxadustat group compared with the placebo or ESA group (MD = 1.65, 95% CI = 1.37, 1.93, *P* < 0.01, *I*^2^ = 92% and MD = 0.16, 95% CI = 0.05, 0.27, *P* < 0.01, *I*^2^ = 0% respectively, Table 2).

### Effects on Iron Metabolism Parameters

In all the studies, IV iron preparations were strictly restricted, and oral iron preparations were generally permitted. As described above, if the studies reported the data for more than one dose, we chose the optimal dose (the most effective dose with the lowest risk of adverse events), such as the high dose (1.50–2.25 mg/kg) in the study of Chen et al. (25) for NDD-CKD patients, 100 mg TIW in the study of Akizawa et al. (24) and mid-range dose (1.5–2.3 mg/kg) in the study of Chen et al. (25) for DD-CKD patients in the roxadustat group to perform the pooled analysis. In the NDD-CKD patients, roxadustat significantly decreased hepcidin, ferritin and transferrin saturation and increased the total iron-binding capacity and transferrin compared with placebo (MD = −19.16, 95% CI = −37.67, −0.65, *P* = 0.04, *I*^2^ = 96%, MD = −50.16, 95% CI = −71.19, −29.13, *P* < 0.01, *I*^2^ = 55%, MD = −2.73, 95% CI = −5.23, −0.23, *P* = 0.03, *I*^2^ =74%, MD = 70.22, 95% CI = 33.86, 106.58, *P* < 0.01, *I*^2^ = 94% and MD = 0.65, 95% CI =0.26, 1.04, *P* < 0.01, *I*^2^ = 95% respectively, Table 2). Total iron was not significantly changed (MD = −2.21, 95% CI = −9.24, 4.81, *P* = 0.54, *I*^2^ = 20%, Table 2). In DD-CKD patients, roxadustat significantly increased the total iron-binding capacity, total iron and transferrin compared with ESA (MD = 42.56, 95% CI = 22.95, 62.18, *P* < 0.01, *I*^2^ = 86%, MD = 16.28, 95% CI = 9.39, 23.17, *P* < 0.01, *I*^2^ = 27% and MD = 0.42, 95% CI = 0.29, 0.54, *P* < 0.01, *I*^2^ = 75% respectively, Table 2). Despite the fact that hepcidin and ferritin were decreased in the roxadustat group, the difference was not significant compared with the levels in the ESA group (MD = −13.62, 95% CI = −35.77, 8.53, *P* = 0.23, *I*^2^ = 77% and MD = −17.46, 95% CI = −61.63, 26.71, *P* = 0.44, *I*^2^ = 73% respectively, Table 2). The rate of oral iron therapy was also decreased, but the difference was not significant (Table 2).

### Effects on Cholesterol Levels

Six studies reported the levels of total cholesterol, low-density lipoprotein (LDL), non-high-density lipoprotein (HDL) and the LDL/HDL ratio (13, 14, 16, 18, 19, 25). However, only the LDL levels data could be pooled and analyzed. The results showed that LDL levels decreased in the roxadustat group compared with the ESA group in DD-CKD patients but no significant change was observed compared with the placebo group in NDD-CKD patients (MD = −18.59, 95% CI = −21.77, −15.42, *P* < 0.01, *I*^2^ = 0% and MD = −16.11, 95% CI = −32.15, −0.07, *P* = 0.05, *I*^2^ = 98%, respectively, Table 2).

### Effects of Roxadustat in Patients With a State of Hyperinflammation

Three studies (15, 18, 26) included in this meta-analysis reported that the EPO treatment effect decreased when the patient was in a state of hyperinflammation (CRP > 4.9 mg/L), whereas the effect of roxadustat did not. However, the data could not be extracted and pooled for analysis.

### Publication Bias

No evidence of publication bias was observed in the outcomes of adverse events (*P* = 0.174 for Begg's test, *P* = 0.260 for Egger's test) in the DD-CKD patients but it was detected in the NDD-CKD patients (*P* = 0.024 for Begg's test, *P* = 0.529 for Egger's test). Regarding the hemoglobin response rate, publication bias was detected for this outcome in the NDD-CKD patients (*P* = 0.573 for Begg's test, *P* = 0.000 for Egger's test) while the funnel plot showed that there was no significant publication bias in the DD-CKD patients (*P* = 0.327 for Begg's test, *P* = 0.579 for Egger's test) (see Supplementary Material).

## Discussion

This meta-analysis evaluated the efficacy and safety of roxadustat vs. placebo for the treatment of anemia in NDD-CKD patients and roxadustat vs. ESA (epoetin alfa or darbepoetin alfa) for the treatment of anemia in DD-CKD patients. The results suggested that roxadustat can be safely used in CKD patients and has an effect similar to that of ESA on increasing hemoglobin. However, the changes in the iron metabolism parameters indicated that the patients may not be receiving the appropriate iron therapy. Therefore, additional clinical trials are still required to further prove whether roxadustat can improve iron metabolism.

Although previous meta-analyses (28–34) have compared the efficacy and safety of roxadustat vs. placebo or ESA, the results showed that roxadustat was more effective than placebo and non-inferior to ESA in correcting anemia in CKD patients. However, evidence for the results is still lacking and several new clinical trials have been conducted to compare roxadustat with placebo or ESA for the treatment of anemia in NDD-CKD or DD-CKD patients. Therefore, we performed this meta-analysis to further investigate the effectiveness and safety of roxadustat therapy for anemia in both the NDD-CKD and DD-CKD patients. In addition, we performed TSA to provide more conservative estimates and more conclusive evidence for the outcomes.

The results showed that there was no significant difference in the total adverse events associated with roxadustat compared with placebo in the NDD-CKD patient group; however, for the DD-CKD patient group, TSA could not confirm this result. We also evaluated the incidence rates of the serious adverse events of hyperkalemia and infection, and no significant differences were found in DD-CKD patients. However, hyperkalemia events were reported more frequently in the roxadustat group in NDD-CKD patients. Although the forest plot showed a significant difference between the roxadustat and placebo groups, the TSA could not confirm this result. Therefore, large sample sizes and long-duration trials are needed to further assess the safety of roxadustat. Since the HIF-PHI pathway is involved in multiple biological processes, such as the upregulation of the erythropoietin gene, regulation of vascular endothelial growth factors (VEGFs) and glycolytic enzymes (1), promotion of tumor metastasis by stimulating epithelial-to-mesenchymal transition (35) and induction of tumor cell invasion (36), safety concerns for HIF stabilizers, including the risk of the development or progression of malignancy, diabetic retinopathy, heart failure, pulmonary hypertension, infection, inflammation, autoimmune disease, kidney fibrosis and polycystic kidney disease, should be carefully considered (37). Recently, the U.S. Food and Drug Administration (FDA) Cardiovascular and Renal Drugs Advisory Committee (CRDAC) voted to recommend not approving roxadustat for anemia in adult CKD patients because of its potential risk of adverse cardiovascular events. Considering that HIF-PHIs are highly likely to exhibit off-target activity in injured kidneys, long observation periods are needed to confirm the safety of HIF-PHIs (10).

Our meta-analysis showed that oral roxadustat was more effective than placebo as a therapy for anemia in NDD-CKD patients, and it was non-inferior to ESA in correcting anemia in DD-CKD patients. Although the change in hemoglobin from baseline was significantly increased in the roxadustat group compared with the ESA group, the hemoglobin response rate was similar in the two groups. It is possible that there was a potential bias in these trials due to the small sample size; thus, future international clinical trials are needed to further evaluate the efficacy of roxadustat vs. ESA.

Roxadustat can activate the HIF signaling pathway and thus increase EPO production (38). HIF2α plays a key role in intestinal iron uptake, iron transport and the use of iron via hepcidin-dependent and hepcidin-independent mechanisms (37, 39). Hepcidin impairs both iron absorption from duodenal enterocytes and iron release from macrophages, where most iron is stored (26). Among the included studies, IV iron preparations were strictly restricted (only in some emergency cases or cases where the iron parameters met the lower limit), and oral iron preparations were generally permitted. Although the decrease in hepcidin may increase iron uptake and iron transport and improve iron utilization, the increase in total iron-binding capacity and transferrin as well as the decrease in ferritin and transferrin saturation among the included patients indicated that the body's iron storage and available iron were decreased after roxadustat treatment in NDD-CKD patients. This result suggests that the present iron therapy may not meet the body iron demand during the treatment. Moreover, in DD-CKD patients, roxadustat did not significantly decrease the values of hepcidin, ferritin, or transferrin saturation or the rate of oral iron therapy compared with ESA. Due to the lack of a stranded iron application in roxadustat clinical studies, it is difficult to evaluate the effect of roxadustat on iron metabolism. Further studies should focus on iron metabolism.

The efficacy of roxadustat in decreasing the levels of LDL in DD-CKD patients may be partly due to the HIF-1-induced activation of Insig-2 and accelerated degradation of 3-hydroxy-3-methylglutaryl (HMG)-CoA reductase (40, 41). However, the effect on other cholesterol level parameters are needs to be further evaluated. Although the treatment effect of roxadustat may not be affected by the state of hyperinflammation, the relationship among those factors requires further analysis.

The publication bias was detected in the outcomes of adverse events and hemoglobin response rate in NDD-CKD patients through Begg's and Egger's test. However, due to the limited number of included studies, we cannot perform suitable subgroup or sensitivity analyses. Although publication bias was not detected in these two outcomes in DD-CKD patients, the funnel plots of them were apparently asymmetric, which indicated that publication bias may also exist in these outcomes. Therefore, further research is needed to assess the potential publication bias more accurately and achieve a more reliable conclusion.

### Limitations

There are several limitations of our meta-analysis. First, four phase 2 trials were included, which decreased the level of evidence. Second, due to the limited amount of data, we could not analyze possible confounding factors by subgroup or sensitivity analyses. Third, the treatment durations were different among the included studies, resulting in between-study heterogeneity. Fourth, the included studies were conducted with a relatively small sample size; therefore, large sample size trials are required to further evaluate whether the results are reliable. Fifth, this meta-analysis only included the published literature, but not the literature supplemented by other resources, which decreased the level of evidence. Finally, long-term follow-up results were still lacking. Long observation periods are needed to further investigate the long-term effect of roxadustat and to confirm whether HIF-PHIs exhibit off-target activity in injured kidneys. Therefore, further high-quality RCTs are needed to confirm or refute this finding.

## Conclusion

In conclusion, our meta-analysis demonstrated that roxadustat could be safely used in CKD patients. Oral roxadustat was more effective than placebo treating anemia in NDD-CKD patients, and it was non-inferior to ESAs in correcting anemia in DD-CKD patients. However, the changes in iron metabolism parameters may indicate that the patients were not receiving appropriate oral iron therapy, and additional clinical trials are needed to further demonstrate whether roxadustat can optimize iron metabolism.

## Data Availability Statement

The original contributions presented in the study are included in the article/Supplementary Material, further inquiries can be directed to the corresponding author/s.

## Author Contributions

CL, ZF, and JJ conceived the study, participated in the design, collected the data, performed the statistical analyses, and drafted the manuscript. CL and ZF contributed equally to this work. KC, XG, ZM, CS, and GS performed statistical analyses and helped draft the manuscript. QH, GC, and XC critically revised the manuscript for important intellectual content. XS collected the data, performed the statistical analyses, and helped revise the manuscript critically for important intellectual content. XS and QH are both guarantors, and they contributed equally to this work. All authors read and approved the final manuscript.

## Conflict of Interest

The authors declare that the research was conducted in the absence of any commercial or financial relationships that could be construed as a potential conflict of interest.

## Publisher's Note

All claims expressed in this article are solely those of the authors and do not necessarily represent those of their affiliated organizations, or those of the publisher, the editors and the reviewers. Any product that may be evaluated in this article, or claim that may be made by its manufacturer, is not guaranteed or endorsed by the publisher.
