# Supplementary material for: Safety and Efficacy of Roxadustat for Anemia in Patients With Chronic Kidney Disease: A Meta-Analysis and Trial Sequential Analysis
Source: Front Med (Lausanne). 2021 Aug 31;8:724456. doi: 10.3389/fmed.2021.724456 (PMC8438137; doi:10.3389/fmed.2021.724456)
Supplement: Supplementary file 1 [file Data_Sheet_1.pdf]

PRISMA 2020 Checklist

Section/topic	Item No	Checklist item	Reported on Page Number/Line Number	Reported on Section/Paragraph
**TITLE**				
Title	1	Identify the report as a systematic review.	3/75-77	Title
**ABSTRACT**				
Abstract	2	See the PRISMA 2020 for Abstracts checklist (Table 2).	3/78-104	Abstract
**INTRODUCTION**				
Rationale	3	Describe the rationale for the review in the context of existing knowledge.	4/116-129	Introduction
Objectives	4	Provide an explicit statement of the objective(s) or question(s) the review addresses.	4/130-137	Introduction
**METHODS**				
Eligibility criteria	5	Specify the inclusion and exclusion criteria for the review and how studies were grouped for the syntheses.	4-5/152-161	Methods
Information sources	6	Specify all databases, registers, websites, organisations, reference lists and other sources searched or consulted to identify studies. Specify the date when each source was last searched or consulted.	4/143-146	Methods
Search strategy	7	Present the full search strategies for all databases, registers and websites, including any filters and limits used.	4/146-151	Methods
Selection process	8	Specify the methods used to decide whether a study met the inclusion criteria of the review, including how many reviewers screened each record and each report retrieved, whether they worked independently, and if applicable, details of automation tools used in the process.	5/153-161	Methods
Data collection process	9	Specify the methods used to collect data from reports, including how many reviewers collected data from each report, whether they worked independently, any processes for obtaining or confirming data from study investigators, and if applicable, details of automation tools used in the process.	5/162-170	Methods
Data items	10a	List and define all outcomes for which data were sought. Specify whether all results that were compatible with each outcome domain in each study were sought (e.g. for all measures, time points, analyses), and if not, the methods used to decide which results to collect.	5/156-159	Methods
	10b	List and define all other variables for which data were sought (e.g. participant and intervention characteristics, funding sources). Describe any assumptions made about any missing or unclear information.	NA	Methods

Study risk of bias assessment	11	Specify the methods used to assess risk of bias in the included studies, including details of the tool(s) used, how many reviewers assessed each study and whether they worked independently, and if applicable, details of automation tools used in the process.	5/165-170	Methods
Effect measures	12	Specify for each outcome the effect measure(s) (e.g. risk ratio, mean difference) used in the synthesis or presentation of results.	5/172-175	Methods
Synthesis methods	13a	Describe the processes used to decide which studies were eligible for each synthesis.	NA	Methods
	13b	Describe any methods required to prepare the data for presentation or synthesis, such as handling of missing summary statistics, or data conversions.	NA	Methods
	13c	Describe any methods used to tabulate or visually display results of individual studies and syntheses.	5/179-190	Methods
	13d	Describe any methods used to synthesize results and provide a rationale for the choice(s). If meta-analysis was performed, describe the model(s), method(s) to identify the presence and extent of statistical heterogeneity, and software package(s) used.	5/171-190	Methods
	13e	Describe any methods used to explore possible causes of heterogeneity among study results.	NA	Methods
	13f	Describe any sensitivity analyses conducted to assess robustness of the synthesized results.	NA	Methods
Reporting bias assessment	14	Describe any methods used to assess risk of bias due to missing results in a synthesis (arising from reporting biases).	5/176-178	Methods
Certainty assessment	15	Describe any methods used to assess certainty (or confidence) in the body of evidence for an outcome.	NA	Methods
**RESULTS**				
Study selection	16a	Describe the results of the search and selection process, from the number of records identified in the search to the number of studies included in the review, ideally using a flow diagram.	6/192-201	Results
	16b	Cite studies that met many but not all inclusion criteria (‘near-misses’) and explain why they were excluded.	Figure1	Results
Study characteristics	17	Cite each included study and present its characteristics.	Table1	Results
Risk of bias in studies	18	Present assessments of risk of bias for each included study.	Figure 2	Results
Results of individual studies	19	For all outcomes, present, for each study: (a) summary statistics for each group (where appropriate) and (b) an effect estimate and its precision (e.g. confidence/credible interval), ideally using structured tables or plots.	Table 1	Results

Results of syntheses	20a	For each synthesis, briefly summarise the characteristics and risk of bias among contributing studies.	6/203-206	Results
	20b	Present results of all statistical syntheses conducted. If meta-analysis was done, present for each the summary estimate and its precision (e.g. confidence/credible interval) and measures of statistical heterogeneity. If comparing groups, describe the direction of the effect.	6-8/207-281	Results
	20c	Present results of all investigations of possible causes of heterogeneity among study results.	NA	Results
	20d	Present results of all sensitivity analyses conducted to assess the robustness of the synthesized results.	NA	Results
Reporting biases	21	Present assessments of risk of bias due to missing results (arising from reporting biases) for each synthesis assessed.	8/282-290	Results
Certainty of evidence	22	Present assessments of certainty (or confidence) in the body of evidence for each outcome assessed.	NA	Results
**DISCUSSION**				
Discussion	23a	Provide a general interpretation of the results in the context of other evidence.	8-10/292-370	Discussion
	23b	Discuss any limitations of the evidence included in the review.	10/371-382	Discussion
	23c	Discuss any limitations of the review processes used.	10/371-382	Discussion
	23d	Discuss implications of the results for practice, policy, and future research.	10-383-389	Discussion
**OTHER INFORMATION**				
Registration and protocol	24a	Provide registration information for the review, including register name and registration number, or state that the review was not registered.	Registration number: CRD42020160014	Methods
	24b	Indicate where the review protocol can be accessed, or state that a protocol was not prepared.	PROSPERO database	Methods
	24c	Describe and explain any amendments to information provided at registration or in the protocol.	PROSPERO database	Methods
Support	25	Describe sources of financial or non-financial support for the review, and the role of the funders or sponsors in the review.	10/390-392	NA
Competing interests	26	Declare any competing interests of review authors.	11/394-395	NA
Availability of data, code and other materials	27	Report which of the following are publicly available and where they can be found: template data collection forms; data extracted from included studies; data used for all analyses; analytic code; any other materials used in the review.	11/407-408	NA

Table 2 PRISMA 2020 for Abstracts checklist

Section/topic	Item No	Checklist item	Reported on Page Number/Line Number	Reported on Section/Paragraph
**TITLE**				
Title	1	Identify the report as a systematic review.	3/75-77	Title
**BACKGROUND**				
Objectives	2	Provide an explicit statement of the main objective(s) or question(s) the review addresses.	3/79-81	Background
**METHODS**				
Eligibility criteria	3	Specify the inclusion and exclusion criteria for the review.	3/84-86	Methods
Information sources	4	Specify the information sources (e.g. databases, registers) used to identify studies and the date when each was last searched.	3/82-84	Methods
Risk of bias	5	Specify the methods used to assess risk of bias in the included studies.	NA	Methods
Synthesis of results	6	Specify the methods used to present and synthesize results.	NA	Methods
**RESULTS**				
Included studies	7	Give the total number of included studies and participants and summarise relevant characteristics of studies.	3/87-88	Results
Synthesis of results	8	Present results for main outcomes, preferably indicating the number of included studies and participants for each. If meta-analysis was done, report the summary estimate and confidence/credible interval. If comparing groups, indicate the direction of the effect (i.e. which group is favoured).	3/88-98	Results
**DISCUSSION**				
Limitations of evidence	9	Provide a brief summary of the limitations of the evidence included in the review (e.g. study risk of bias, inconsistency and imprecision).	3/101-102	Conclusion
Interpretation	10	Provide a general interpretation of the results and important implications.	3/99-101	Conclusion
**OTHER**				
Funding	11	Specify the primary source of funding for the review.	NA	NA
Registration	12	Provide the register name and registration number.	NA	NA

Please leave this space alone as it will be supplemented by the editorial office when needed.
