# Supplementary material for: Safety and Efficacy of Roxadustat for Anemia in Patients With Chronic Kidney Disease: A Meta-Analysis and Trial Sequential Analysis
Source: Front Med (Lausanne). 2021 Aug 31;8:724456. doi: 10.3389/fmed.2021.724456 (PMC8438137; doi:10.3389/fmed.2021.724456)
Supplement: Supplementary file 2 [file Data_Sheet_1.docx]

***Supplementary Material***

**S1.** Funnel plots of lgrr in adverse events.

**S2.** Funnel plots of lgrr in hemoglobin response rate.

**B**

**A**

**D**

**C**

**S1.** Funnel plots of lgrr in adverse events. **(A, B)** Funnel plots of lgrr in adverse events in NDD-CKD patients (P = 0.024 for Begg’s test, P = 0.529 for Egger’s test). **(C, D)** Funnel plots of lgrr in adverse events in DD-CKD patients (P = 0.174 for Begg’s test, P = 0.260 for Egger’s test). Abbreviations: CKD, chronic kidney disease; DD, dialysis-dependent; NDD, non-dialysis-dependent.

**B**

**A**

**D**

**C**

**S2.** Funnel plots of lgrr in hemoglobin response rate. **(A, B)** Funnel plots of lgrr in hemoglobin response rate in NDD-CKD patients (P = 0.573 for Begg’s test, P = 0.000 for Egger’s test). **(C, D)** Funnel plots of lgrr in hemoglobin response rate in DD-CKD patients (P = 0.327 for Begg’s test, P = 0.579 for Egger’s test). Abbreviations: CKD, chronic kidney disease; DD, dialysis-dependent; NDD, non-dialysis-dependent.
